# Infiltration of dendritic cells and antigen uptake in the muscle after injection of HIV-1 Env gp120 in adjuvant

**DOI:** 10.1186/1742-4690-9-S2-O16

**Published:** 2012-09-13

**Authors:** F Liang, KJ Sandgren, H Fausther-Bovendo, D O'hagan, A Seubert, E De Gregorio, S Rao, N Sullivan, RA Seder, RA Koup, K Loré

**Affiliations:** 1 Vaccine Research Center, NIH, Bethesda, MD, USA; 2 Novartis Vaccines and Diagnostics, Cambridge, MD, USA; 3 Karolinska Institutet, Stockholm, Sweden

## Background

Most vaccines are delivered into the muscle although it contains very few potent antigen presenting cells, such as dendritic cells (DCs) that are critical for driving adaptive immune responses. Understanding the early mechanisms that dictate vaccine responses and why some adjuvants like the oil-in-water emulsion MF59 are shown to be more potent than alum is important for the design of new vaccines. Here, we investigated the recruitment of immune cells to the vaccine injection site and uptake of a clinically relevant HIV-1 envelope glycoprotein (Env) and MF59 in a non-human primate (NHP) model.

## Methods

Rhesus macaques received intramuscular injections of either fluorescently-labeled Env gp120 alone or together with MF59 in the deltoid and quadriceps muscles. Donor-matched injections of PBS and MF59 alone served as controls. At 24-72 hrs, blood, muscle and lymph nodes were sampled for flow cytometry and confocal microscopy.

## Results

There was a robust infiltration into the muscle of multiple immune cells by MF59+/-Env. CD66abce+ neutrophils were most frequent followed by CD14+ monocytes and CD11c+ myeloid DCs. CD123+ plasmacytoid DCs which do not normally reside in muscle, were also recruited by MF59. Internalization of Env and MF59 was readily detectable in all DC subsets both in the muscle and in the draining lymph nodes. Although injection of Env alone did not lead to cell infiltration, the few resident DCs showed efficient Env uptake. Groups receiving Env together with distinctly different adjuvants (alum and TLR7 ligand) are underway.

## Conclusion

MF59 as an adjuvant leads to significant influx of cells that efficiently engulf vaccine protein antigen. Antigen/adjuvant carrying DC subsets appear early in the lymph nodes draining the injection site. As NHP DC subsets are similar to humans, this offers a powerful model that can yield data to be translated into optimizing future vaccine formulations and delivery strategies.

